# NCOA4-Mediated Ferritinophagy Contributes to Iron Overload-Driven Ferroptosis of Senescent Myoblasts in Mice

**DOI:** 10.3390/cells15161434

**Published:** 2026-08-10

**Authors:** Yan Huang, Zhen Qi, Chuan Chen, Zhihua Yu

**Affiliations:** Shanghai Geriatric Institute of Chinese Medicine, Shanghai University of Traditional Chinese Medicine, 365 South Xiangyang Road, Shanghai 200031, China

**Keywords:** ferritinophagy, ferroptosis, iron overload, aging, sarcopenia

## Abstract

Sarcopenia is an age-related pathological syndrome characterized by progressive and generalized loss of skeletal muscle mass and function, with muscle atrophy representing its cardinal pathological hallmark. Ferroptosis, an iron-dependent regulated cell death, has been implicated in the pathogenesis of muscle atrophy; however, the precise role of iron dysregulation in sarcopenia remains incompletely understood. In the present study, we identified ferroptosis in D-galactose (D-gal)-induced senescent myoblasts, as evidenced by elevated intracellular iron levels and lipid peroxidation, increased malondialdehyde (MDA) content, and upregulated expression of prostaglandin endoperoxide synthase 2 (PTGS2), 4-hydroxynonenal (4-HNE), and long-chain acyl-CoA synthetase 4 (ACSL4), accompanied by diminished glutathione peroxidase 4 (GPX4), SLC7A11 (xCT), and glutathione (GSH) levels, as well as pronounced mitochondrial damage. Notably, treatment with the iron chelator deferoxamine (DFO) significantly attenuated senescence-associated ferroptosis. Moreover, D-gal-induced senescence in myoblasts was accompanied by reduced ferritin expression and elevated nuclear receptor coactivator 4 (NCOA4) levels, both of which were reversed by autophagy inhibition with 3-methyladenine (3-MA) or NCOA4 knockdown, suggesting that NCOA4-mediated ferritinophagy is involved in senescence-induced iron overload and ferroptosis. Furthermore, senescent myoblasts exhibited increased reactive oxygen species (ROS) generation and mitochondrial impairment, which were attributed to cytosolic iron overload-mediated upregulation of mitoferrin 2 (Mfrn2), thereby promoting mitochondria iron import. Finally, pharmacological inhibition of iron overload or ferroptosis by DFO or ferrostatin-1 (Ferr-1) effectively ameliorated skeletal muscle atrophy and functional decline in aged sarcopenia mice. Collectively, these findings elucidate the mechanistic basis of sarcopenia and highlight potential therapeutic avenues targeting iron dysregulation and ferroptosis.

## 1. Introduction

Human aging is frequently characterized by a progressive decline in organ function. Skeletal muscle, the body’s largest tissue mass, undergoes gradual reduction beginning around the third decade of life. By the age of 70, muscle mass decreases by approximately 40%, while muscle strength diminishes to nearly 35% of its youthful peak [1,2]. Age-associated deterioration manifests clinically as sarcopenia, a progressive syndrome defined by the loss of skeletal muscle mass and function. Beyond compromising physical performance, sarcopenia markedly impairs activities of daily living in older adults, thereby elevating the risk of falls, disability, and mortality [3]. Moreover, sarcopenia acts as a catalyst for systemic comorbidities; the associated decline in basal metabolic rate, coupled with increased adiposity and bone mineral loss, fosters a metabolic milieu conducive to chronic disease, such as diabetes, hypertension, osteoporosis, coronary artery disease, obesity, and cognitive impairment [4].

Iron is an indispensable micronutrient involved in oxygen transport, hematopoiesis, and immune regulation [5,6]. Notably, skeletal muscle accounts for approximately 15% of the total body iron stores. Accumulating evidence indicates that physiological aging is associated with intramuscular iron overload. In experimental models exhibiting iron overload, marked elevations in muscle ferritin levels coincide with elevated malondialdehyde (MDA), a lipid peroxidation byproduct. These perturbations correlate with distinct phenotypic changes, including attenuated muscle mass, reduced myofiber cross-sectional area, diminished contractile strength, and compromised locomotor performance. Mechanistically, iron overload-induced skeletal muscle atrophy and function loss are strongly implicated in oxidative stress-mediated injury and mitochondrial dysfunction [6,7,8,9].

Ferroptosis is a recently characterized, iron-dependent regulatory cell death modality driven by the accumulation of lipid peroxides, the accumulation of reactive oxygen species (ROS), and dysregulated iron metabolism [10,11]. Intracellular iron overload facilitates this process by providing free Fe^2+^, which triggers the Fenton reaction to generate cytotoxic hydroxyl radicals. This cascade amplifies oxidative stress, precipitating massive ROS production and overwhelming lipid peroxidation repair mechanisms, thereby executing the ferroptosis program [12,13,14].

In our previous findings, sarcopenic-like aged SAMP8 mice exhibited marked iron overload and lipid peroxidation in skeletal muscle, and excessive iron exposure elicited the accumulation of lipid peroxidation byproducts and initiated ferroptosis in myoblasts, which was then effectively rescued by inhibition with either ferrostatin-1 (Fer-1) or the iron chelator deferoxamine (DFO) [15]. Nevertheless, the precise molecular circuitry linking age-related iron overload to ferroptosis in myoblasts remains to be fully elucidated. Therefore, the present study aimed to explore the regulatory mechanisms of age-dependent iron accumulation, investigate the signaling pathways of iron overload-induced ferroptosis in myoblasts, and assess the potential involvement of iron overload in sarcopenia progression.

## 2. Materials and Methods

### 2.1. Cell Culture and Differentiation

C2C12 myoblasts (Cell Bank, Shanghai Institute for Biological Science, Shanghai, China. Accession No. SCSP-505) were cultured in the growth medium (GM), comprising Dulbecco’s modified Eagle’s medium (DMEM, 25 mM glucose, Thermo Fisher Scientific, Waltham, MA, USA) supplemented with 10% fetal bovine serum (FBS, Thermo Fisher Scientific, Waltham, MA, USA) and 1% penicillin–streptomycin (Thermo Fisher Scientific, Waltham, MA, USA). When cells reached 80% confluence, the medium was switched to differentiation medium (DM), consisting of DMEM supplemented 2% horse serum (Thermo Fisher Scientific, Waltham, MA, USA) and 1% penicillin–streptomycin. Five days after differentiation, cells were used for experiments. All cells were cultured in an incubator at 37 °C in a humidified atmosphere of 95% air and 5% CO_2_, and the culture medium was changed every other day.

### 2.2. Cell Treatments

D-gal was dissolved in PBS and diluted to the appropriate concentration at the time of use. To test the effect of D-gal on C2C12 cells, cells were incubated with different doses of D-gal (20 or 40 mg/mL), and 40 mg/mL D-gal treatment was selected as the effective experimental condition for senescence induction. The cells were treated with DFO (100 µM) for cytoplasmic iron chelation, with ferrostatin-1 (Ferr-1, 10 µM) to inhibit ferroptosis, and with 3-MA (25 µM) to inhibit autophagy as per the experimental requirements.

### 2.3. Cell Viability Assay

Cell viability was measured using the AlamarBlue Cell Viability Reagent (Invitrogen, Carlsbard, CA, USA) according to the manufacturer’s instructions. In brief, after cells were cultured in a medium supplemented with 10% AlamarBlue for 2 h, 100 µL of the culture medium was transferred to 96-well plates and quantified spectrophotometrically with a microplate reader (SpectraMax i3x, Molecular Devices, San Jose, CA, USA) at wavelengths of 570 nm and 650 nm. The culture media from non-cell-seeded disks were similarly analyzed as blank controls.

### 2.4. Cell Senescence Assay

For SA-β-Gal staining, the C2C12 cell was fixed with the fixative solution at room temperature for 30 min. Cells were then washed with PBS for three times, and the SA-β-Gal staining working solution was added before incubating overnight at 37 °C without CO_2_. Cells were observed under the microscope (CX43 Biological Microscope, Olympus, Tokyo, Japan) and photographed.

For fluorescence detection of β-gal, the CellEvent^TM^ Senescence Green Detection Kit (Invitrogen, Carlsbad, CA, USA) was used. After washing with PBS, the cells were fixed with 4% paraformaldehyde for 10 min at room temperature. Then the cells were washed with 100 µL of 1% BSA in PBS per well to remove the fixation solution, and the prewarmed work solution with diluted green probe was added. The cells were then incubated for 2 h at 37 °C without CO_2_, covered with plastic film to prevent moisture loss and light. After incubation, the working solution was removed, and the cells were washed by PBS. Images were taken images using the microscope (CX43 Biological Microscope, Olympus, Tokyo, Japan).

### 2.5. Lipid Peroxidation Assay

The treated cells were trypsinized to obtain cell suspension and incubated with a 10 µM BODIPY^TM^ 581/591C11 fluorescence probe (Thermo Scientific, Invitrogen, Waltham, MA, USA) for 30 min in a humidified incubator (at 37 °C, 5% CO_2_). Following three washes with PBS, the lipid peroxidation levels were measured and analyzed by flow cytometry (Cytomics FC 500, BECKMAN, Brea, CA, USA).

### 2.6. Transmission Electron Microscopy

The cells and muscles were collected and fixed with 2.5% glutaraldehyde at room temperature in the dark for 30 min, then washed several times with PBS, dehydrated in graded concentrations of ethanol, embedded in SPI-Pon 812 Epoxy Resin (Sigma-Aldrich, St. Louis, MO, USA), and polymerized for 48 h at 60 °C. Sections were cut with a diamond knife (Daitome, Nidau, Switzerland) using a Leica Ultramicrotome (EM UC7, Leica, Hamburg, Germany) and stained with both methanolic uranyl acetate and lead citrate before viewing in a Tecnai G2 20 Twin (FEI, Thermo Fisher Scientific, Waltham, MA, USA) transmission electron microscope. For each sample, five fields of view were randomly selected and the images were captured. Images were evaluated by two independent electron microscopy specialists who were blinded to the information of the specimen.

### 2.7. Detection of Iron in Cells and Tissues

As previously described [16], FerroOrange (Dojindo, Kumamoto, Japan) and Mito-FerroGreen (Dojindo, Kumamoto, Japan) were used to detect intracellular Fe^2+^ and mitochondrial Fe^2+^ respectively. For FerroOrange staining, the cells were incubated with 1μM FerroOrange in Hank’s balanced salt solution (HBSS) for 30 min at 37 °C and observed under confocal laser scanning microscopy (CX43 Biological Microscope, Olympus, Tokyo, Japan). For Mito-FerroGreen staining, the cells were incubated with 5 μM Mito-FerroGreen (Dojindo, Kumamoto, Japan) working solution for 30 min at 37 °C, and the presence of mitochondrial Fe^2+^ was observed under a fluorescence microscope (CX43 Biological Microscope, Olympus, Tokyo, Japan).

The non-heme iron of cells and muscle tissues was measured using a colorimetric Iron Assay kit (Abcam, Cambridge, UK). Following the manufacturer’s instructions, the tissue was homogenized in iron assay buffer on ice and centrifuged at 16,000 *g* for 10 min to collect the supernatant. The supernatant was then mixed with iron reducer in a 96-well plate and incubated at 37 °C for 30 min. Subsequently, the iron probe was added, mixed, and incubated at 37 °C for 60 min protected from light, and the plate was then measured immediately at 593 nm on a colorimetric microplate reader (SpectraMax i3x, Molecular Devices, CA, USA).

### 2.8. NADPH Assay

Nicotinamide adenine dinucleotide phosphate (NADPH) content was measured using a fluorometric NADP/NADPH Assay Kit (Abcam, Cambridge, UK). Cells or muscle tissues were homogenized in lysis buffer, and the homogenate was then centrifuged at 2500 *g* for 5 min. The collected supernatant was mixed with NADPH extraction solution in a 96-well plate and incubated at room temperature for 15 min, before being neutralized with NADP Extraction Solution. The NADPH reaction mixture was then added, mixed, and incubated at room temperature for 2 h in the dark, and the fluorescence was immediately measured at Ex/Em = 540/590 nm on a microplate reader (SpectraMax i3x, Molecular Devices, CA, USA).

### 2.9. Measurement of MDA and GSH Content

The levels of malondialdehyde (MDA) and glutathione (GSH) in cells and tissues were detected using a Lipid Peroxidation MDA Assay Kit (Beyotime, Shanghai, China) and a GSH and GSSG Assay Kit (Beyotime, Shanghai, China), respectively. Both measurements strictly followed the manufacturer’s instructions, and the contents are indicated as μmol/g protein.

### 2.10. Quantitative Real-Time PCR Analysis

Total RNA was isolated from cells and tissues using TRIZOL reagent (Invitrogen, Carlsbard, CA, USA). An equivalent amount of each RNA sample was converted to cDNA using the PrimeScript RT kit (Takara, Shiga, Japan). PCR amplification was performed on a real-time PCR machine (ABI 7500, Thermo Fisher Scientific, Waltham, MA, USA) using a SYBR Green PCR kit (SYBR Premix EX Taq, TaKaRa, Shiga, Japan) with GAPDH (glyceraldehyde-3-phosphate dehydrogenase) used for normalization. The data were analyzed using the ^2−ΔΔ^Ct method. The primers for RT-qPCR are listed below:

Mouse Ptgs2-F: 5′-CTGCGCCTTTTCAAGGATGG-3′.

Mouse Ptgs2-R: 5′-GGGGATACACCTCTCCACCA-3′.

Mouse Fbxo32-F: 5′-AGAGAGGCAGATTCGCAAGCGT-3′.

Mouse Fbxo32-R: 5′-TGCAAAGCTGCAGGGTGACCC-3′.

Mouse Trim63-F: 5′-ACCTGCTGGTGGAAAACATC-3′.

Mouse Trim63-R: 5′-CTTCGTGTTCCTTGCACATC-3′.

Mouse P21-F: 5′-CCTGGTGATGTCCGACCTG-3′.

Mouse P21-R: 5′-CCATGAGCGCATCGCAATC-3′.

Mouse P16-F: 5′-CGCAGGTTCTTGGTCACTGT-3′.

Mouse P16-R: 5′-TGTTCACGAAAGCCAGAGCG-3′.

Mouse Il6-F: 5′-TAGTCCTTCCTACCCCAATTTCC-3′.

Mouse Il6-R: 5′-TTGGTCCTTAGCCACTCCTTC-3′.

Mouse Il1b-F: 5′-GAAATGCCACCTTTTGACAGTG-3′.

Mouse Il1b-R: 5′-TGGATGCTCTCATCAGGACAG-3′.

Mouse Tnfa-F: 5′-CCCTCACACTCAGATCATCTTCT-3′.

Mouse Tnfa-R: 5′-GCTACGACGTGGGCTACAG-3′.

Mouse Col1a1-F: 5′-GCTCCTCTTAGGGGCCACT-3′.

Mouse Col1a1-R: 5′-CCACGTCTCACCATTGGGG-3′.

Mouse Col3a1-F: 5′-CTGTAACATGGAAACTGGGGAAA-3′.

Mouse Col3a1-R: 5′-CCATAGCTGAACTGAAAACCACC-3′.

### 2.11. Western Blot Analysis

Cells or tissues were lysed in radio immunoprecipitation assay (RIPA) lysis buffer with protease inhibitor cocktail for 30 min on ice. Then the lysates were centrifuged at 12,000 *g* for 10 min, and the protein in the supernatant was collected. Protein concentrations were measured by performing a bicinchoninic (BCA) assay. Each protein lysate (20 μg) was resolved via sodium dodecyl sulfate polyacrylamide gel electrophoresis (SDS-PAGE) using 10% gels, and proteins were then transferred to polyvinylidene difluoride (PVDF) membranes (Millipore, Burlington, MA, USA). Nonspecific interactions were blocked with 5% skim milk for 1 h, and membranes were then probed with specific primary antibodies to P16 (1:1000, Abcam, Cambridge, UK), P21 (1:1000, Abcam, Cambridge, UK), γ-H2AX (1:1000, Abcam, Cambridge, UK), 4-hydroxynonenal (1:1000, Abcam, Cambridge, UK), glutathione peroxidase 4 (Gpx4, 1:1000, Abcam, Cambridge, UK), SLC7A11 (xCT, 1:1000, Abcam, Cambridge, UK), long-chain acyl-CoA synthetase 4 (ACSL4, 1:1000, Santa Cruz Biotechnology, Santa Cruz, CA, USA), transferrin receptor 1 (TrfR1, 1:1000, Abcam, Cambridge, UK), ferroportin 1 (FPN1, 1:1000, Invitrogen, Carlsbad, CA, USA), ferritin heavy chain (FTH, 1:1000, Abcam, Cambridge, UK), ferritin light chain (FTL, 1:1000, Abcam, Cambridge, UK), nuclear receptor coactivator 4 (NCOA4, 1:1000; Invitrogen, Carlsbad, CA, USA) and GAPDH (1:1000, Cell Signaling Technology, Danvers, MA, USA) overnight at 4 °C. HRP-conjugated secondary antibodies (1:3000, Genscript, Nanjing, China) were used at 37 °C for 1 h. The bound antibodies were visualized using an enhanced chemiluminescence detection system (Millipore, Burlington, MA, USA) and quantified using Image-Pro Plus 6.0 software (Media Cybernetics, Rockville, MD, USA).

### 2.12. Mitochondrial ROS Measurement

C2C12 myoblasts and the senescent C2C12 myoblasts induced by D-gal were treated with or without DFO for 24 h. As previously described [16], to measure mitochondrial superoxide levels, the cells were incubated with 5 μM MitoSOX probe (Beyotime, Shanghai, China) for 30 min at 37 °C, washed with PBS 3 times, and then stained with Hoechst 33,258 (Beyotime, Shanghai, China). The cells were observed and photographed using a fluorescence microscope (CX43 Biological Microscope, Olympus, Tokyo, Japan), and the images were analyzed using Image-Pro Plus 6.0 software (Media Cybernetics, Rockville, MD, USA).

### 2.13. ATP Measurement

The cellular ATP levels were measured using a firefly luciferase-based enhanced ATP assay Kit according to the manufacturer’s protocol (Beyotime, Shanghai, China). Briefly, the cells were lysed by the lysis buffer and centrifuged at 12,000 *g* for 5 min. The supernatant was collected and then added into the substrate solution. After reacting in the room temperature for 3 min, the luminescence was recorded using a microplate reader (SpectraMax i3x, Molecular Devices, CA, USA).

### 2.14. Mitochondrial Membrane Potential Measurement

The mitochondrial membrane potential (ΔΨ m) in cells was measured by a mitochondrial membrane potential assay kit with JC-1. In depolarized mitochondria, the red fluorescent aggregates in normal mitochondria transform into green fluorescent monomers. The degree of mitochondrial dysfunction was evaluated in terms of red/green fluorescence intensity ratio. According to the manufacturer’s protocol (Beyotime, Shanghai, China), the cells were stained by the probe for 20 min at 37 °C and then stained with Hoechst 33,258 after washing with PBS 3 times. The images were photographed using a fluorescence microscope (CX43 Biological Microscope, Olympus, Tokyo, Japan) and analyzed using Image-Pro Plus 6.0 software (Media Cybernetics, Rockville, MD, USA).

### 2.15. Mitochondrial Morphology Assessment

The cellular mitochondrial morphology was observed after staining by the MitoTracker Red CMXRos for 30 min at 37 °C, according to the manufacturer’s protocol (Beyotime, Shanghai, China). Images were then captured under a fluorescence microscope (CX43 Biological Microscope, Olympus, Tokyo, Japan). The mitochondria were divided into the following three categories as previously described [16,17]: category I—elongated mitochondria organized in a tubular network; category II—large, dotted mitochondria evenly distributed all over the cytosol; category III—totally fragmented mitochondria around the nucleus.

### 2.16. Small Interfering RNA (siRNA) and Transfection

NCOA4 and Mfrn2-targeting siRNAs (siNCOA4 and siMfrn2) or non-targeting control siRNAs (siNC) were synthesized by GenePharm (Shanghai, China). The sequences are listed below (sequences of siMfrn2 as previously described [16]). The cells were transiently transfected with 20 µM siNCOA4, siMfrn2, or siNC using Lipofectamine 3000 (Invitrogen, Carlsbad, CA, USA) according to the manufacturer’s instructions. The treatments were initiated at 48 h post-transfection, and the cells were harvested for the total cellular protein or mRNA.

Mouse siNCOA4-F: 5′-GGGCUGAACAGCAAAUUAATT-3′.

Mouse siNCOA4-R: 5′-UUAAUUUGCUGUUCAGCCCTT-3′.

Mouse siMfrn2-F: 5′-CCAUUCACUUCAUGACCUATT-3′.

Mouse siMfrn2-R: 5′-UAGGUCAUGAAGUGAAUGGTT-3′.

### 2.17. Animals and Treatments

This study complied with the ethical standards of the institutional Animal Care and Use Committee (IACUC) and was approved by the Ethics Committee of Shanghai Yishang Biotechnology Co., Ltd. (Shanghai, China) (Approval No. YS-JL-001; 2 September 2024). The wild-type C57BL/6JRj male mice were purchased from Shanghai Laboratory Animal Center (SLAC) Co., Ltd. (Shanghai, China). All the mice were kept in an SPF-grade animal facility at 24 °C with a relative humidity of 50–60%, and in a light/dark cycle of 12 h/12 h. Young (3 months old, as a negative control) and aged (22 months old) mice were used for the subsequent in vivo experiments. A total of 45 aged mice were randomly divided into control (Old), DFO, and Ferr-1 groups (15 mice for each group). For treatments, DFO (5 µg/TA) or Ferr-1 (0.5 µg/TA) diluted in 50 µL of saline was injected into the TA muscles of the mice using a 29-gauge needle and a 1 mL syringe (Becton, Dickinson and Company, Franklin Lakes, NJ, USA). The needle was inserted deep into the TA muscle longitudinally toward the knee and DFO or Ferr-1 was injected along the length of the muscle every other day for 2 consecutive months. Mice in the control group were injected with 50 µL of saline. After treatments, the mice underwent grip strength tests, treadmill exhaustion tests, and muscle force analysis. They were then sacrificed, with their TA muscles collected for subsequent testing.

### 2.18. Grip Strength Test

The mice were held, facing the bar of a grip strength meter (YLS-13A, Jinan Yiyan Technology Development Co., Ltd., Jinan, China), while their forearms were gently restrained by the experimenter. After the mouse grasped the bar, when its unrestrained forepaw was brought into contact with the bar of the grip strength meter, the mouse was gently pulled away from the device. The grip strength meter then measured the maximal force applied before the animal released the bar. The maximal force was measured 10 times for each mouse.

### 2.19. Treadmill Exhaustion Test

A treadmill exhaustion test was performed using a treadmill Exhaustion Device (YLS-10B, Jinan Yiyan Technology Development Co., Ltd., Jinan, China) for both young and old mice with or without treatment. The treadmill running period started with an adaptation period of 10 m/min for 2 min before an increase of 2 m/min every 2 min until fatigue response was initiated, and it was terminated when mice no longer responded to five consecutive fatigue stimuli (1.5 mA current for up to 5 s). Upon fatigue initiation, mice were quickly removed from the treadmill running lane. Treadmill running time and distance were recorded and calculated for all mice.

### 2.20. In Vivo Muscle Force Analysis

In vivo TA muscle force analysis was performed with a 1300 A 3-in-1 whole animal system (Aurora Scientific Inc., Aurora, ON, Canada), as previously described [18]. Briefly, mice were anesthetized via tribromoethanol (25 mg/g body weight) intraperitoneal injection and kept warm via a heat lamp. The distal portion of the TA muscle and its tendon were dissected from the mice, and the tendon was tied with a bread silk suture to the lever arm of the force transducer. Then, the contractile force was measured by DMC software (version 5, Aurora scientific). During the process, the TA muscle was constantly moistened with pre-warmed 37 °C Ringer’s buffer. For each treatment, three independent experiments were performed.

### 2.21. Immunological Staining and Histomorphometric Analysis

Tissues were fixed and embedded in O.C.T. compound, frozen in liquid nitrogen-cooled isopentane, stored at −80 °C, and cut into 10 µm thick cryosections at −20 °C. The sections of tibialis anterior (TA) muscles for immunohistochemical staining were incubated with primary antibody to Glutathione peroxidase 4 (Gpx4, 1:200, Abcam, Cambridge, UK) or nuclear receptor coactivator 4 (NCOA4, 1:200; Invitrogen, Carlsbad, CA, USA) overnight at 4 °C, and with secondary anti-goat IgG (Sigma-Aldrich, St. Louis, MO, USA) for 1 h at room temperature. A streptavidin–horseradish peroxidase (HRP) detection system was used to detect the antigen, and sections were visualized with diaminobenzidine tetrahydrochloride. For immunofluorescence staining of differentiated myotubes, cells were fixed with 4% paraformaldehyde for 15 min, and the anti-MYHC (Myosin Heavy Chain, Sigma, Merck, Darmstadt, Germany, 1:1000) was used as the primary antibody, with the Alexa Fluor 488-labeled anti-mouse IgG antibody (R&D Systems, Minneapolis, MN, USA) being the second antibody. For immunofluorescence staining of myofiber, sections of the tibialis anterior (TA) muscles were processed using rabbit anti-mouse Lamin A primary antibody (1:200, Sigma-Aldrich, St. Louis, MO, USA) and Alexa Fluor 488-labeled anti-mouse IgG antibody (R&D Systems, Minneapolis, MN, USA) and DAPI (1 mg/mL, Roche, Basel, Switzerland). For immunofluorescence staining of MHC types, sections of TA muscles were incubated with the following primary antibodies for 2 h: BA-F8 for MHC type I (1:50), SC-71 for MHC type II a (1:600), and BF-F3 for MHC type II b (1:100) (Developmental Studies Hybridoma Bank, Iowa City, IA, USA). They were then incubated with the following secondary antibodies for 1 h: Alexa Fluor 350 IgG2b (1:500), Alexa Fluor 488 IgG1 (1:500), and Alexa Fluor 555 IgM (1:500) (Invitrogen). Stained sections were visualized using a fluorescence microscope (CX43 Biological Microscope, Olympus, Tokyo, Japan) and analyzed using Image-Pro Plus 6.0 software (Media Cybernetics, Rockville, MD, USA).

### 2.22. Statistical Analysis

All data were obtained from three or more experiments, and values are presented as means ± standard deviation. Statistical analyses were performed using a one-way analysis of variance (ANOVA), followed by the Student–Newman–Keul post hoc test. *p* < 0.05 was considered to indicate a statistically significant difference.

## 3. Results

### 3.1. Iron Overload and Ferroptosis Were Triggered in Senescent Myoblasts Induced by D-Gal and Skeletal Muscles from Aged Mice

**Figure 1:**
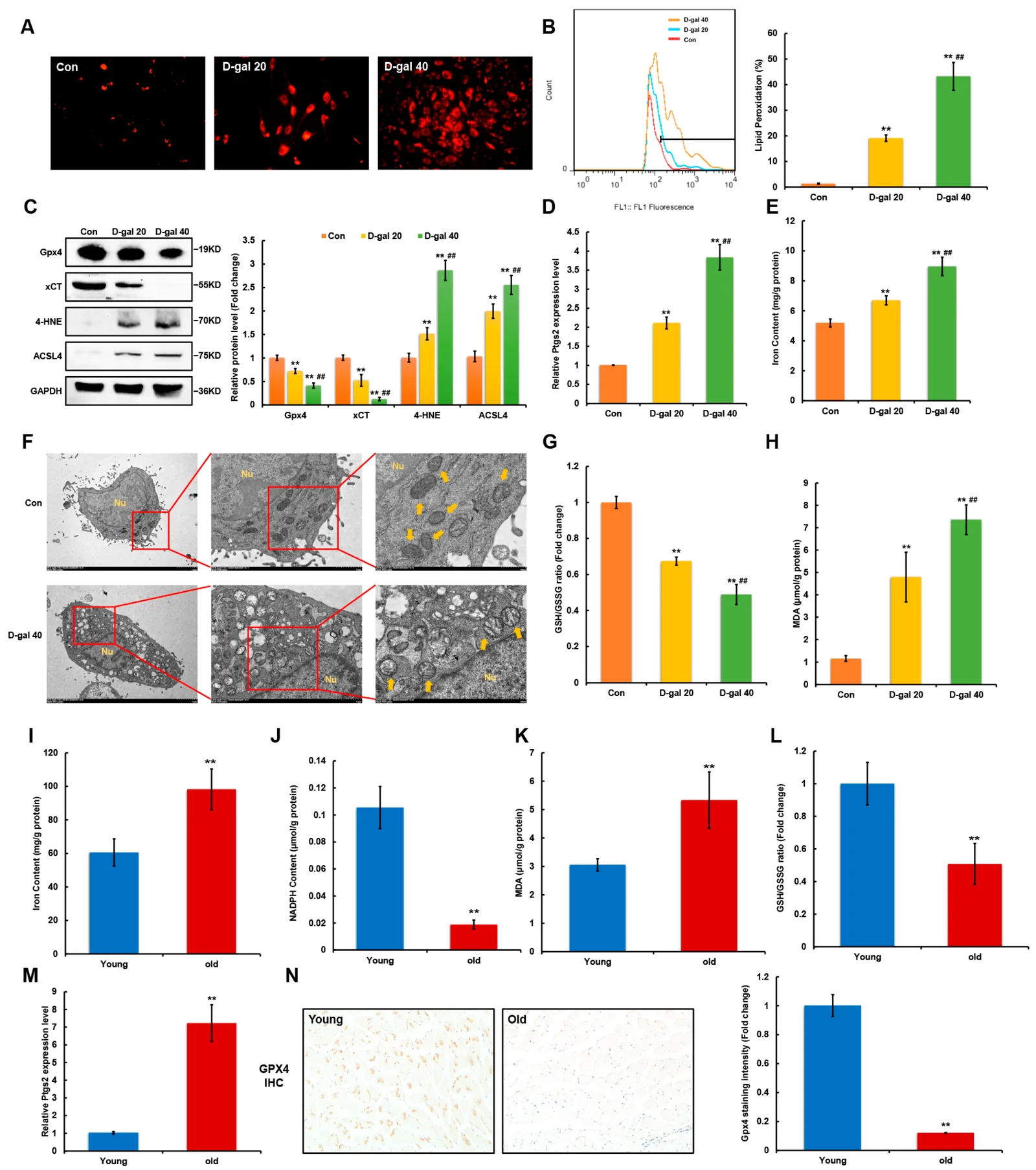
Iron overload and ferroptosis occurred in D-gal-induced senescent myoblasts. (**A**) Representative images of FerroOrange-stained myoblasts showing intracellular Fe^2+^ levels in the absence or presence of 20 mg/mL or 40 mg/mL D-gal treatment (magnification: 200×). (**B**) Representative images of C11-BODIPY-stained C2C12 cells showing lipid peroxidation 24h after treatment with or without 20 mg/mL or 40 mg/mL D-gal. Bar graphs showing relative levels of lipid peroxidation in the indicated cells. *n* = 3 independent experiments (** *p* < 0.01 vs. control group, ^##^ *p* < 0.01 vs. 20 mg/mL D-gal-treated group). (**C**) Representative immunoblots and quantitative analysis showing the expression levels of Gpx4, xCT, 4-HNE and ACSL4 in non-treated or 20 mg/mL or 40 mg/mL D-gal-treated C2C12 myoblasts (** *p* < 0.01 vs. control group, ^##^ *p* < 0.01 vs. 20 mg/mL D-gal-treated group). *n* = 3 independent experiments. (**D**) Ptgs2 mRNA level and (**E**) iron content in myoblasts treated in the absence or presence of 20 mg/mL or 40 mg/mL D-gal (** *p* < 0.01 vs. control group, ^##^ *p* < 0.01 vs. 20 mg/mL D-gal-treated group). *n* = 3 independent experiments. (**F**) Representative TEM images of myoblasts showing the ultrastructure of mitochondria in the absence or presence of 20 mg/mL or 40 mg/mL D-gal (magnification: 4000×, 10,000× and 20,000×. ‘Nu’ indicates nuclear and arrows indicate mitochondria). (**G**) Glutathione (GSH) and (**H**) malondialdehyde (MDA) levels in non-treated and 20 mg/mL or 40 mg/mL D-gal-treated myoblasts (** *p* < 0.01 vs. control group, ^##^ *p* < 0.01 vs. 20 mg/mL D-gal-treated group). *n* = 3 independent experiments. Bar graphs showing iron (**I**), NADPH (**J**), MDA (**K**), GSH (**L**) and Ptgs2 mRNA (**M**) levels in the muscle tissues of 2-month-old (Young) and 24-month-old (Old) C57BL/6J mice. *n* = 6 per group, (**N**) Representative IHC images and quantitative analysis of Gpx4 staining in muscle sections of 2-month-old and 24-month-old C57BL/6J mice. ** *p* < 0.01 vs. young group. *n* = 6 per group.

In previous studies, D-gal has been shown to induce senescence in C2C12 myoblasts [19,20]. To establish a senescent myoblast model, C2C12 cells were treated with varying concentrations of D-gal. Cell viability was assessed, and the impact of D-gal on cellular senescence was evaluated by SA-β-gal staining. The results demonstrated that D-gal treatment led to a concentration-dependent decrease in C2C12 viability (Supplementary Figure S1) and a concomitant increase in the number of SA-β-gal-positive cells (Supplementary Figure S2A). Furthermore, the mRNA and protein expression levels of the senescent markers (P16, P21 and γ.H2AX) were upregulated to varying degrees following exposure to different concentrations of D-gal (Supplementary Figure S2B,C). In addition, a significant downregulation of the late myogenic marker (myosin heavy chain, MyHC) was observed, accompanied by a marked increase in the expression of muscle atrophy-related genes (Fbxo32 and Trim63) in response to D-gal treatment (Supplementary Figure S2). Collectively, these findings indicated that D-gal-induced C2C12 senescence and impaired myofiber differentiation in a concentration-dependent manner. Accordingly, a concentration of 40 mg/mL D-gal was selected as the senescence-inducing dose for all subsequent experiments. To determine whether senescence promotes iron overload and ferroptosis in myoblasts, C2C12 cells were exposed to different concentrations of D-gal for 24 h. The result of FerroOrange staining revealed a significant accumulation of intracellular Fe^2+^ in D-gal-induced senescent C2C12 cells (Figure 1A), with iron overload intensifying proportionally to the D-gal concentration (Figure 1E). Concurrently, lipid peroxidation increased significantly in a concentration-dependent manner (Figure 1B). Moreover, the expression of ferroptosis marker PTGS2 (mRNA), the lipid peroxidation marker ACSL4, and 4-hydroxynonenal (4-HNE) were markedly upregulated (Figure 1C,D), whereas the protein levels of glutathione peroxidase 4 (GPX4) and xCT were substantially decreased (Figure 1C). Biochemical analyses further confirmed the presence of lipid oxidative stress, as cellular GSH levels were reduced (Figure 1G) while MDA levels were elevated following D-gal treatment (Figure 1H). Consistent with these observations, transmission electron microscopy revealed mitochondria with diminished or absent cristae and a ruptured outer membrane in D-gal-induced senescent C2C12, representing the characteristic morphological features compatible with ferroptosis (Figure 1F). To validate these findings in vivo, tibialis anterior (TA) muscles were harvested from aged C57BL/6 mice (24 months old) to assess senescence-associated ferroptosis. A significant increase in iron content was detected in TA muscles of aged mice, indicating aging-related iron overload (Figure 1I). Additionally, the level of the antioxidant NADPH (Figure 1J) and GSH (Figure 1L) were markedly decreased, whereas the MDA levels were significantly elevated (Figure 1K), reflecting enhanced lipid oxidation in aged muscle tissues. Concomitantly, the upregulation of Ptgs2 mRNA (Figure 1M) and the downregulation of GPX4 protein (Figure 1N) provided further evidence of ferroptosis in aged skeletal muscle. Taken together, these results demonstrate that myoblast senescence could induce muscular iron overload and ferroptosis in both in vitro and in vivo models.

### 3.2. Decreasing Intracellular Iron Levels Attenuated Ferroptosis in Senescent Myoblasts Induced by D-Gal

**Figure 2:**
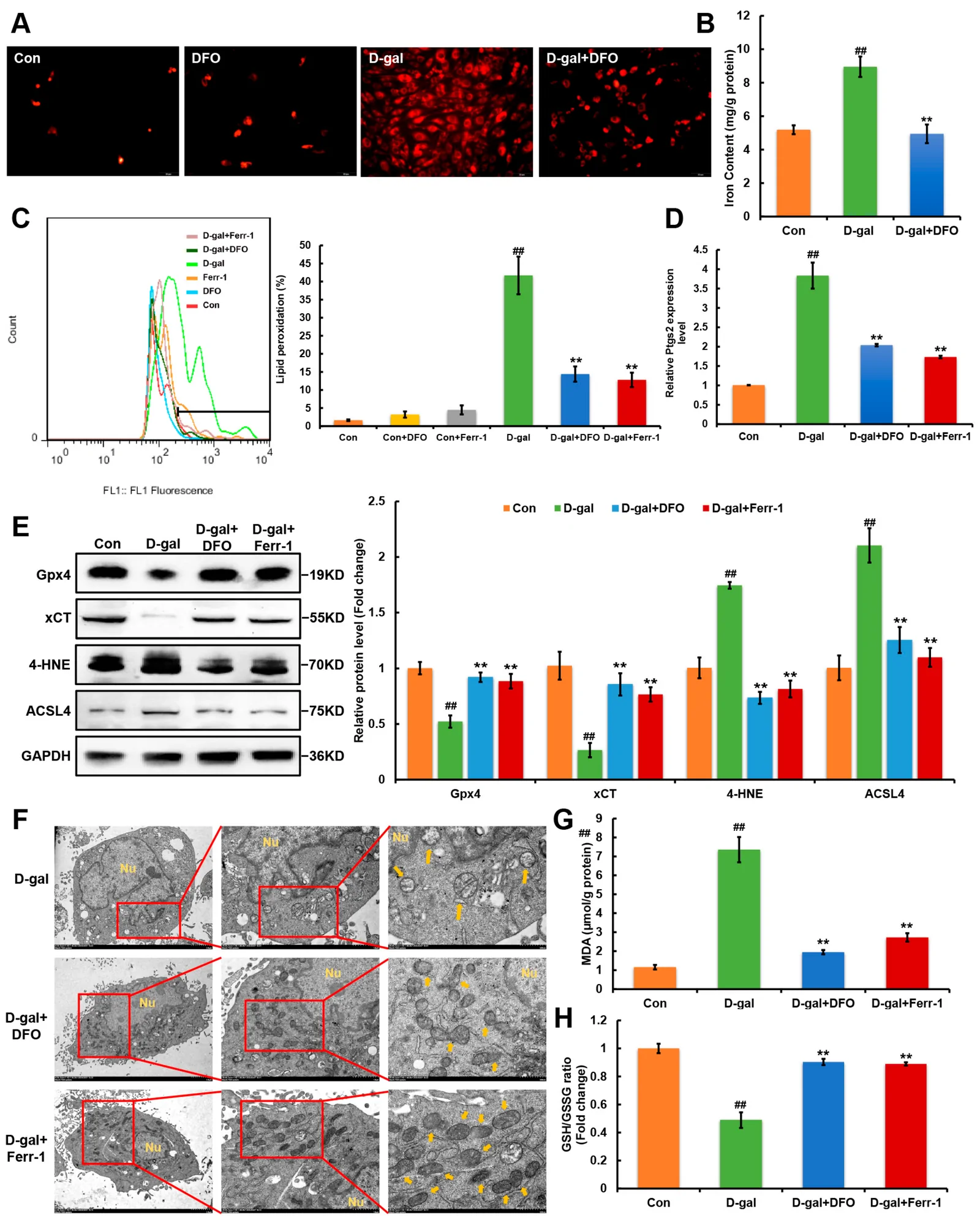
The iron chelator deferoxamine (DFO) alleviated iron overload and ferroptosis in D-gal-induced senescent myoblasts. (**A**) Representative images of FerroOrange-stained control or DFO-treated senescent myoblasts induced by D-gal showing intracellular Fe^2+^ levels (magnification: 200×). (**B**) Iron content in control or DFO-treated D-gal-induced senescent myoblasts. *n* = 3 independent experiments. (**C**) Representative images of C11-BODIPY-stained control, DFO or Ferr-1-treated C2C12 cells in the absence or presence of D-gal treatment. Bar graphs showing relative levels of lipid peroxidation in the indicated cells. *n* = 3 independent experiments. (**D**) Ptgs2 mRNA level in control, DFO or Ferr-1-treated D-gal-induced senescent myoblasts. *n* = 3 independent experiments. (**E**) Representative immunoblots and quantitative analysis showing the expression levels of Gpx4, xCT, 4-HNE and ACSL4 in control, DFO- or Ferr-1-treated D-gal-induced senescent myoblast. *n* = 3 independent experiments. (**F**) Representative TEM images of D-gal-induced senescent myoblasts showing the ultrastructure of mitochondria in the absence or presence of DFO and Ferr-1 treatment (magnification: 4000×, 10,000× and 20,000×. ‘Nu’ indicates nuclear and arrows indicate mitochondria). Bar graphs showing MDA (**G**) and GSH (**H**) levels in D-gal-induced senescent myoblasts treated with or without DFO and Ferr-1. ** *p* < 0.01 vs. D-gal-treated group, ^##^ *p* < 0.01 vs. control group. *n* = 3 independent experiments.

To determine the role of iron in senescent myoblasts, deferoxamine (DFO), a potent iron chelator, was employed to deplete intracellular iron. It was found that DFO treatment effectively reversed the elevated intracellular Fe^2+^ levels observed in D-gal-induced senescent C2C12 cells (Figure 2A,B). Furthermore, ferrostain-1 (Ferr-1), a canonical ferroptosis inhibitor, served as a positive control to validate the involvement of iron in ferroptosis. Similar to the effects of Ferr-1, DFO administration significantly attenuated lipid peroxidation (Figure 2C), Ptgs2 mRNA expression (Figure 2D), 4-HNE and ACSL4 protein levels (Figure 2E), and MDA content (Figure 2G). Conversely, DFO treatment restored GPX4 and xCT protein levels (Figure 2D) and replenished GSH content (Figure 2H) in D-gal-induced senescent C2C12 cells, collectively indicating that iron depletion suppressed ferroptosis. Moreover, paralleling the protective effects of Ferr-1, DFO treatment preserved mitochondrial structural integrity, as evidenced by transmission electron microscopy, which revealed clearly defined mitochondrial cristae and an intact outer membrane (Figure 2F). Thus, DFO-mediated reduction in intracellular iron attenuated ferroptosis in D-gal-induced senescent myoblasts.

### 3.3. Senescence-Induced Intracellular Iron Overload Triggered Ferritinophagy

**Figure 3:**
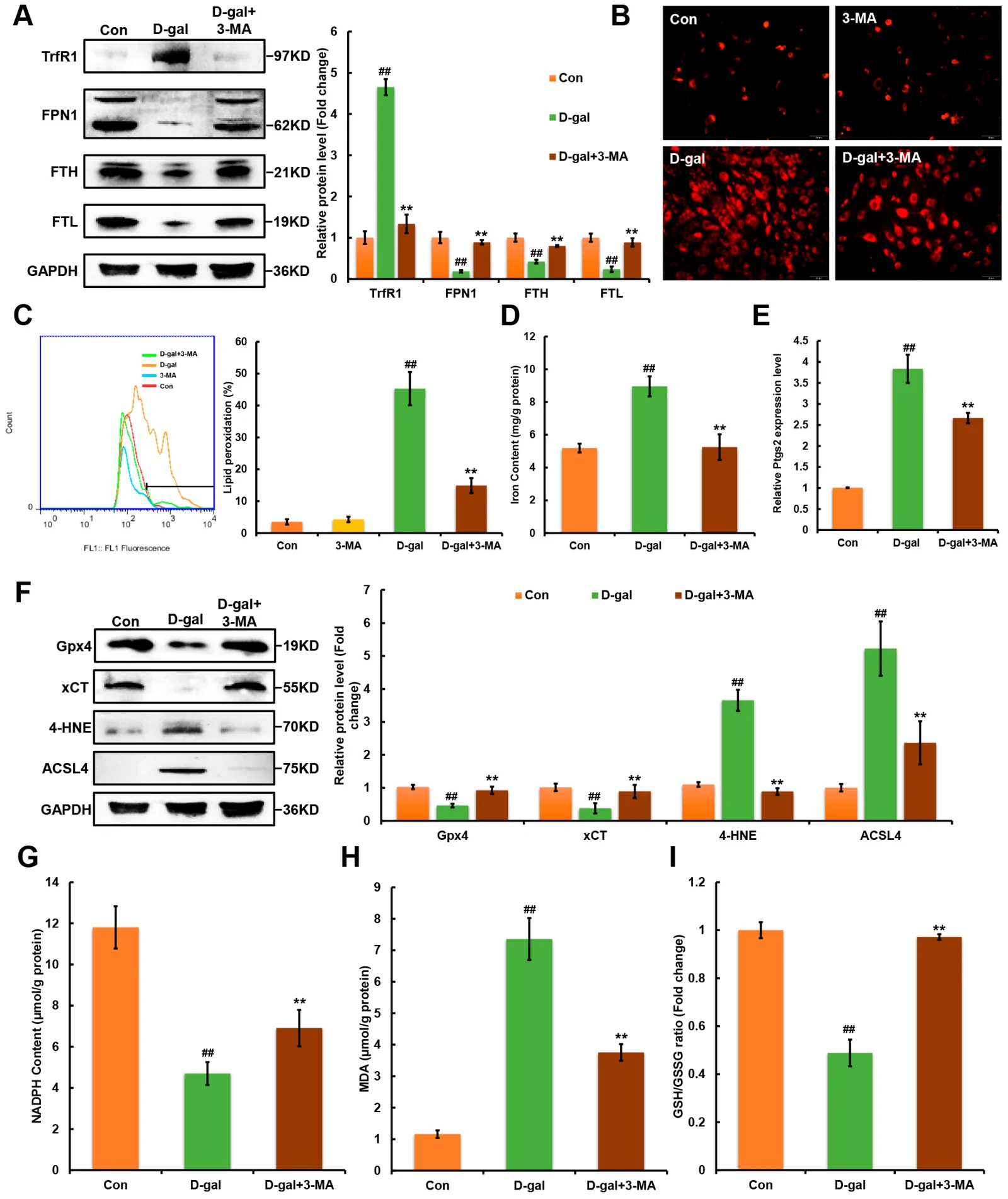
Autophagy was involved in intracellular iron overload and ferroptosis in D-gal-induced senescent myoblast. (**A**) Representative immunoblots and quantitative analysis showing the expression levels of TfR1, FPN1, FTH and FTL in control or D-gal-induced senescent myoblasts treated with or without 3-MA. *n* = 3 independent experiments. (**B**) Representative images of FerroOrange-stained control or D-gal-induced senescent myoblasts treated with or without 3-MA (magnification: 200×). (**C**) Representative images of C11-BODIPY-stained control or 3-MA-treated C2C12 cells in the absence or presence of D-gal treatment. Bar graph showing relative levels of lipid peroxidation in the indicated cells. *n* = 3 independent experiments. Bar graph showing Iron content (**D**) and Ptgs2 mRNA levels (**E**) in control or D-gal-induced senescent myoblasts treated with or without 3-MA. *n* = 3 independent experiments. (**F**) Representative immunoblots and quantitative analysis showing the expression levels of Gpx4, xCT, 4-HNE and ACSL4 in control and 3-MA-treated D-gal-induced senescent myoblasts. *n* = 3 independent experiments. Bar graphs showing NADPH content (**G**), MDA (**H**) and GSH (**I**) levels in control or D-gal-induced senescent myoblasts treated with or without 3-MA. ** *p* < 0.01 vs. D-gal-treated group, ^##^ *p* < 0.01 vs. control group. *n* = 3 independent experiments.

Ferritinophagy, a selective autophagic process, mediates ferritin degradation and subsequent release of free iron [21,22]. To elucidate the involvement of ferritinophagy in senescence-associated iron overload, we assessed the protein levels of key iron-regulatory factors in D-gal-induced senescent C2C12 cells, with or without pharmacological inhibition of autophagy by 3-methyladenine (3-MA). Specifically, we examined proteins governing iron influx and efflux, the transferrin receptor 1 (TfR1) and ferroportin 1 (FPN1), as well as proteins responsible for iron storage, including ferritin heavy chain (FTH) and ferritin light chain (FTL). The results demonstrated that both FTL and FTH were markedly downregulated in D-gal-induced senescent C2C12 cells but restored upon co-treatment with 3-MA. Moreover, 3-MA effectively reversed the D-gal-induced upregulation of TfR1 and downregulation of FPN1 (Figure 3A) and suppressed the senescence-driven accumulation of intracellular Fe^2+^ (Figure 3B,D). Furthermore, 3-MA treatment markedly attenuated lipid peroxidation and ferroptosis, as evidenced by decreased lipid peroxidation (Figure 3C), reduced Ptgs2 mRNA expression (Figure 3E), lower protein levels of 4-HNE and ACSL (Figure 3F), and diminished MDA content (Figure 3H). Conversely, 3-MA administration restored the protein expression of Gpx4 and xCT (Figure 3E) and replenished intracellular NADPH and GSH levels (Figure 3G,I).

### 3.4. NCOA4 Was Involved Senescence-Induced Ferritinophagy

**Figure 4:**
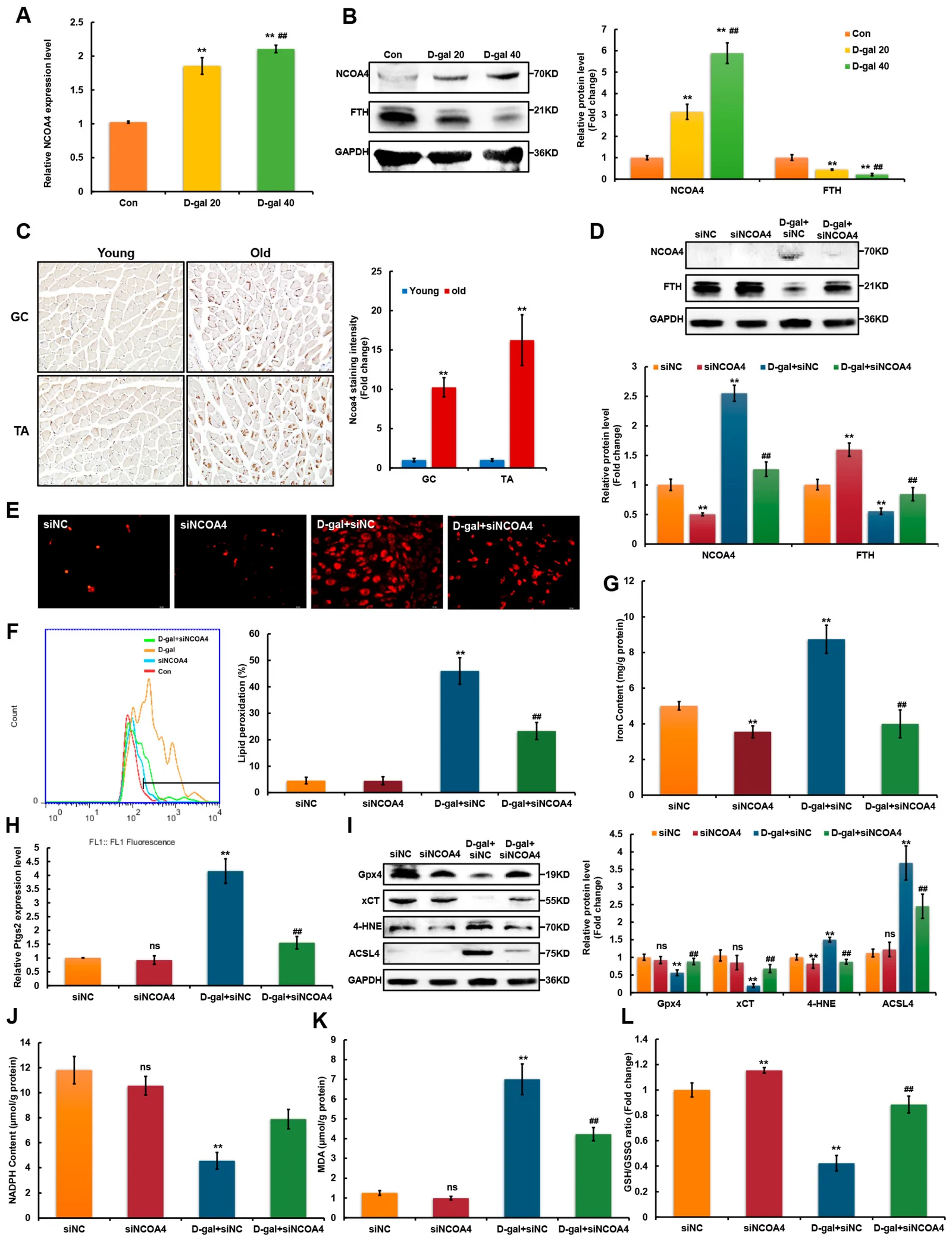
Ferritinophagy via NCOA4-mediated intracellular iron overload and ferroptosis in D-gal-induced senescent myoblasts. NCOA4 mRNA (**A**) and protein (**B**) levels in control and myoblasts treated with 20mg/mL or 40 mg/mL D-gal. ** *p* < 0.01 vs. control group, ^##^ *p* < 0.01 vs. 20 mg/mL D-gal-treated group. *n* = 3 independent experiments. (**C**) Representative IHC images and quantitative analysis of muscle sections of young (2 month) and old (24 month) C57BL/6J mice showing NCOA4 expression (magnification: 200×). ** *p* < 0.01 vs. young group. *n* = 7 per group. (**D**) NCOA4 and FTH protein levels in siNC- or siNCO4-treated control and D-gal-induced senescent myoblasts. ** *p* < 0.01 vs. siNC-treated control group, ^##^ *p* < 0.01 vs. siNC-treated senescent group. *n* = 3 independent experiments. (**E**) Representative images of FerroOrange-stained siNC- or siNCOA4-treated control and D-gal-induced senescent myoblasts showing intracellular Fe^2+^ levels (magnification: 200×). (**F**) Representative images of C11-BODIPY-stained siNC- or siNCOA4-treated C2C12 cells in the absence or presence of D-gal treatment. Bar graph showing the relative levels of lipid peroxidation in the indicated cells. *n* = 3 independent experiments, Bar graphs showing iron content (**G**) and Ptgs2 mRNA levels (**H**) in siNC- or siNCOA4-treated control or D-gal-induced senescent myoblasts. ** *p* < 0.01 vs. siNC-treated control group, ^##^ *p* < 0.01 vs. siNC-treated senescent group. *n* = 3 independent experiments. (**I**) Representative immunoblots and quantitative analysis showing the expression levels of Gpx4, xCT, 4-HNE and ACSL4 in siNC- or siNCO4-treated control and D-gal-induced senescent myoblasts. *n* = 3 independent experiments. Bar graphs showing NADPH content (**J**), MDA (**K**) and GSH (**L**) levels in siNC- or siNCO4-treated control or D-gal-indued senescent myoblasts. ns, *p* > 0.05; ** *p* < 0.01 vs. siNC-treated control group, ^##^ *p* < 0.01 vs. siNC-treated senescent group. *n* = 3 independent experiments.

Nuclear receptor coactivator 4 (NCOA4) has been identified as a key mediator of ferritinophagy [22,23,24]. To elucidate its role in senescence-associated ferritinophagy, we assessed NCOA4 expression in D-gal-treated C2C12 cells. It was found that varying concentrations of D-gal significantly upregulated both NCOA4 mRNA (Figure 4A) and protein (Figure 4B) levels. Consistently, immunohistochemical (IHC) analysis revealed a marked elevation of NCOA4 protein expression in the TA muscles of aged mice (Figure 4C). Functional studies demonstrated that siRNA-mediated knockdown of endogenous NCOA4 (knockdown efficiency is shown in Supplementary Figure S3) effectively reversed the senescence-induced decline in FTH (Figure 4D), thereby reducing intracellular labile iron pools (Figure 4E,G). Consequently, NCOA4 depletion mitigated senescence-induced ferroptosis, as evidenced by decreased levels of lipid peroxidation (Figure 4F), Ptgs2 mRNA (Figure 4H), 4-HNE and ACSL4 protein (Figure 4I), and MDA (Figure 4K), coupled with restored expression of Gpx4 and xCT proteins (Figure 4I) and replenished NADPH (Figure 4J) and GSH (Figure 4L) levels.

### 3.5. Intracellular Iron Overload Led to Elevated Mitochondrial Iron Levels and Subsequent Mitochondrial Dysfunction

**Figure 5:**
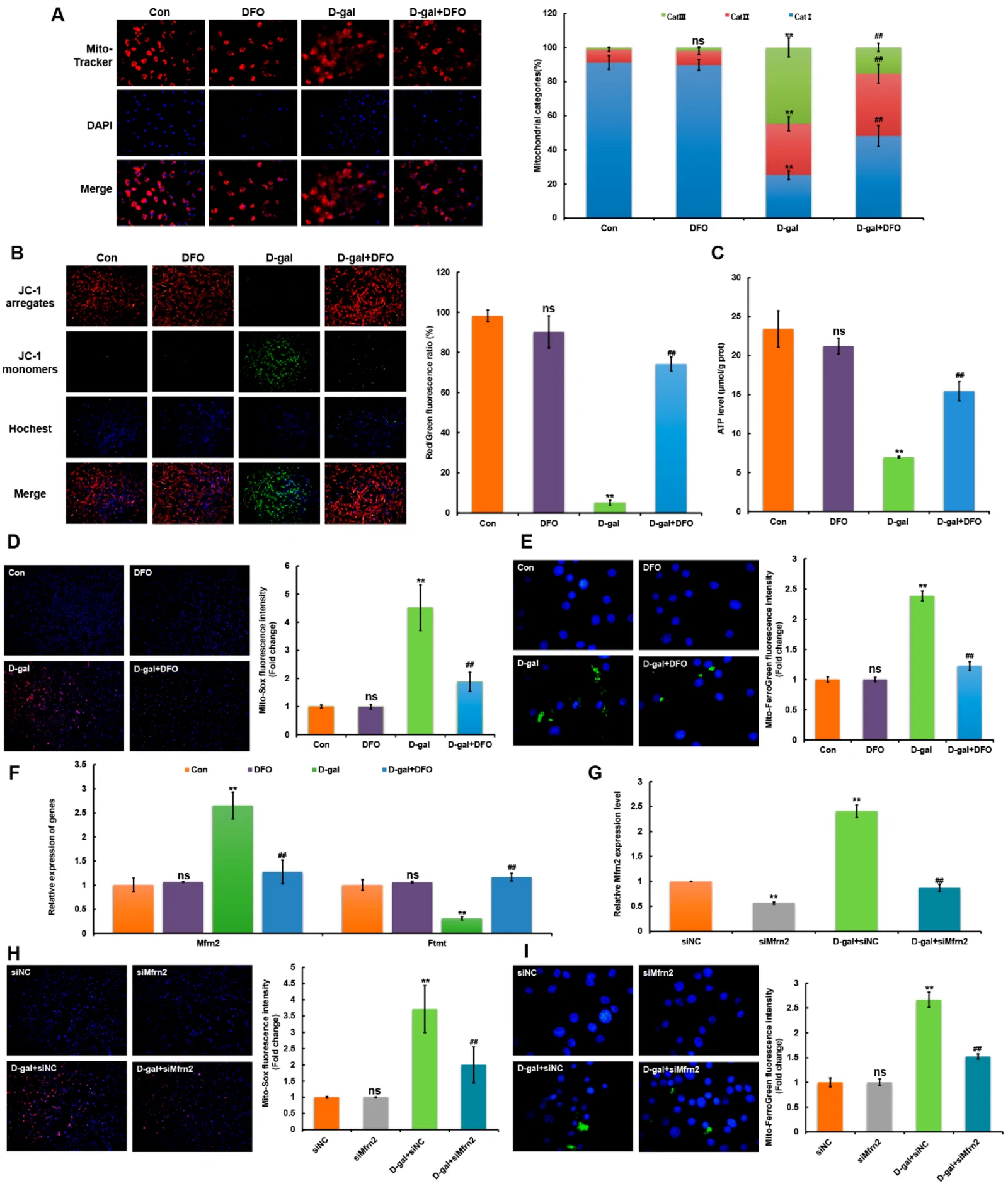
Senescence-induced iron overload triggered mitochondrial dysfunction. (**A**) Representative images and quantification of MitoTracker-stained control and D-gal-induced senescent myoblasts treated with or without DFO showing mitochondrial morphology. Nuclei were counterstained with DAPI (magnification: 200×). *n* = 3 independent experiments. (**B**) Representative images and red/green fluorescence ratio of JC-1-stained control and D-gal-induced senescent myoblasts treated with or without DFO showing mitochondrial membrane potential. Nuclei were counterstained with Hoechst (magnification: 100×). *n* = 3 independent experiments. (**C**) ATP levels in the control and D-gal-induced senescent treated with or without DFO. ns, *p* > 0.05 vs. control group; ** *p* < 0.01 vs. control group, ^##^ *p* < 0.01 vs. D-gal-treated group. *n* = 3 independent experiments. (**D**) Representative images and fluorescence intensity of MitoSox-stained control and D-gal-induced senescent myoblasts treated with or without DFO showing mitochondrial ROS generation. Nuclei were counterstained with Hoechst (magnification: 100×). *n* = 3 independent experiments. (**E**) Representative images and fluorescence intensity of Mito-FerroGreen-stained control and D-gal-induced senescent myoblasts treated with or without DFO showing mitochondrial Fe^2+^. Nuclei were counterstained with Hoechst (magnification: 600×). *n* = 3 independent experiments. (**F**) Mfrn2 and Ftmt mRNA expression in control and D-gal-induced senescent myoblasts treated with or without DFO. ns, *p* > 0.05 vs. control group; ** *p* < 0.01 vs. control group, ^##^ *p* < 0.01 vs. D-gal-treated group. *n* = 3 independent experiments. (**G**) Mfrn2 mRNA levels in siNC- or siMfrn2-treated control and D-gal-induced senescent myoblast. ** *p* < 0.01 vs. siNC-treated control group, ^##^ *p* < 0.01 vs. siNC-treated senescent group. *n* = 3 independent experiments. (**H**) Representative images and fluorescence intensity of MitoSox-stained siNC- or siMfrn2-treated control and D-gal-induced senescent myoblasts showing mitochondrial ROS generation. Nuclei were counterstained with Hoechst (magnification: 100×). ns, *p* > 0.05 vs. siNC-treated control group; ** *p* < 0.01 vs. siNC-treated control group, ^##^ *p* < 0.01 vs. siNC-treated senescent group. *n* = 3 independent experiments. (**I**) Representative images and fluorescence intensity of Mito-FerroGreen-stained siNC- or siMfrn2-treated control and D-gal-induced senescent myoblasts showing mitochondrial Fe^2+^. Nuclei were counterstained with Hoechst (magnification: 600×). ns, *p* > 0.05 vs. siNC-treated control group; ** *p* < 0.01 vs. siNC-treated control group, ^##^ *p* < 0.01 vs. siNC-treated senescent group. *n* = 3 independent experiments.

Mitochondria, as the primary organelles responsible for cellular energy in muscle cells, play a pivotal role in preserving optimal muscle homeostasis and physiological function. As evidenced by the preceding results, the senescence process is accompanied by a marked reduction in mitochondrial cristae and frequent outer membrane rupture in myoblasts. Accordingly, it is plausible to hypothesize that the downstream cellular consequences elicited by senescence-associated iron overload are predominantly mediated by mitochondria. Consistent with previous reports [25,26], cells exhibiting predominantly perinuclear, fragmented mitochondria were categorized as category III, whereas those displaying an extensive network of elongated mitochondria were assigned to category I. In the present study, Mito-Tracker Red staining revealed that D-gal-induced senescence led to pronounced mitochondrial fragmentation and perinuclear clustering in C2C12 cells, which were effectively ameliorated by DFO treatment (Figure 5A). Furthermore, disruption of the mitochondrial membrane potential (MMP), a well-established hallmark of mitochondrial impairment, has been recognized as an early event in ferroptosis [27,28]. JC-1 staining demonstrated that D-gal-induced senescence in C2C12 cells was characterized by enhanced green fluorescence intensity of JC-1 monomers, indicative of a significant loss of MMP, a change that was likewise reversed by DFO administration (Figure 5B). Concomitantly, intracellular ATP levels were markedly reduced in D-gal-induced senescent C2C12 cells but were substantially rescued following DFO intervention, suggesting that intracellular iron overload contributes to senescence-induced mitochondrial dysfunction (Figure 5C).

In addition, an elevated level of mitochondrial ROS was observed in D-gal-induced senescent C2C12 cells (Figure 5D), which could potentially be attributed to increased mitochondrial iron accumulation (Figure 5E), as DFO treatment effectively reversed both mitochondrial iron overload and ROS elevation (Figure 5D,E). Accumulating evidence has demonstrated that iron overload upregulates the mitochondrial iron importer Mfrn2 while downregulating the iron-storage protective protein Ftmt, thereby leading to toxic accumulation of labile iron within mitochondria. Excess mitochondrial iron drives Fenton reactions and lipid peroxidation, thus directly triggering ferroptosis [29,30,31]. Consistent with these findings, the present study demonstrated that D-gal-induced senescence resulted in upregulated Mfrn2 and downregulated Ftmt mRNA expression, alterations that were effectively rescued by DFO treatment (Figure 5F). These results suggested that the dysregulation of Ftmt and Mfrn2 expression was mediated by cytosolic iron overload, which promotes iron influx while suppressing storage capacity, ultimately leading to elevated mitochondrial Fe^2+^ levels. Moreover, functional assays indicated that knockdown of Mfrn2 (Figure 5G) via RNA interference (RNAi) significantly attenuated mitochondrial ROS (Figure 5H) and labile iron levels (Figure 5I). Collectively, these findings demonstrated that D-gal-induced cytosolic iron overload enhanced mitochondrial iron uptake and ROS generation, thereby culminating in mitochondrial dysfunction and ultimately promoting ferroptosis through lipid peroxidation in C2C12 cells.

### 3.6. Inhibition of Iron Overload or Ferroptosis Alleviated Age-Induced Skeletal Muscle Atrophy in Mice

**Figure 6:**
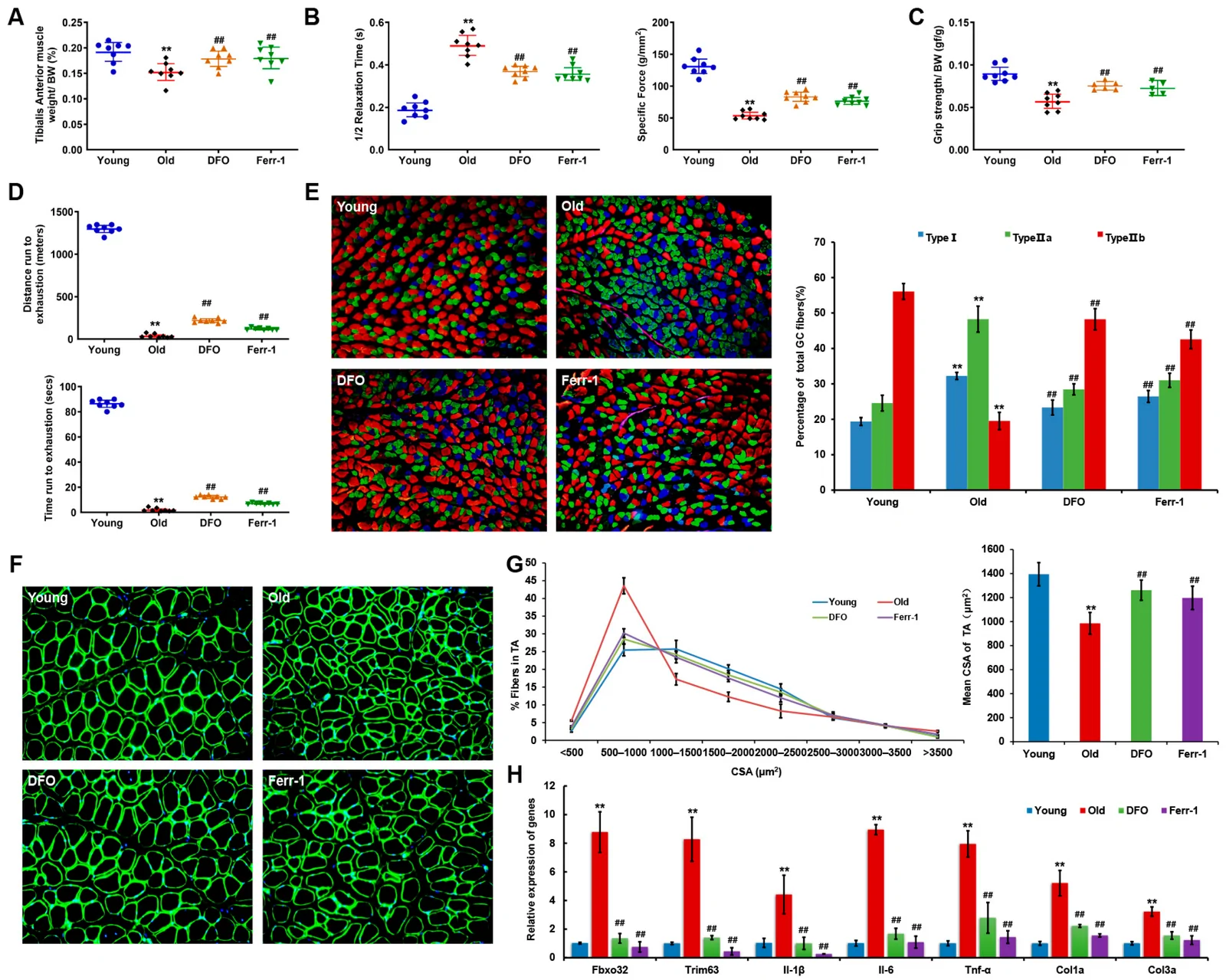
DFO and Ferr-1 ameliorated sarcopenic status in aged C57BL/6J mice. (**A**) TA muscle mass in young and old C57BL/6J mice under different treatments. ** *p* < 0.01 vs. the young group, ^##^ *p* < 0.01 vs. the old group. *n* = 8 per group. (**B**) Muscle contractile properties of TA muscles in young and old C57BL/6J mice under different treatments. ** *p* < 0.01 vs. the young group, ^##^ *p* < 0.01 vs. the old group. *n* = 8 per group. (**C**) Grip strength of TA muscles in young and old C57BL/6J mice under different treatments. ** *p* < 0.01 vs. the young group, ^##^ *p*< 0.01 vs. the old group. *n* = 8 per group. (**D**) Treadmill running distance and time to exhaustion for young and old C57BL/6J mice under different treatments. ** *p* < 0.01 vs. the young group, ^##^ *p* < 0.01 vs. the old group. *n* = 8 per group. (**E**) Representative images and fluorescence intensity of immunofluorescence staining for type I (blue), type II a (green), and type II b (red) myofibers in TA muscles showing the respective percentage of each muscle fiber type. ** *p* < 0.01 vs. the young group, ^##^ *p* < 0.01 vs. the old group. *n* = 8 per group. (**F**) Representative images of immunostaining for laminin (green) in TA muscles of young and old C57BL/6J mice under different treatments (magnification: 200×). (**G**) Bar graphs showing the distribution and mean cross-sectional area (CSA) of myofibers in TA muscles. ** *p* < 0.01 vs. the young group, ^##^ *p* < 0.01 vs. the old group. *n* = 8 per group. (**H**) Relative expression levels of muscle atrophy markers (Fbxo32 and Trim63), senescence markers (P16 and P21), inflammation cytokines (Il1b and Tnfa) and fibrosis-related genes (Col1a1 and Col3a1) in TA muscles. ** *p* < 0.01 vs. the young group, ^##^ *p* < 0.01 vs. the old group. *n* = 6 per group.

To further validate the role of iron overload and ferroptosis in the skeletal muscles during aging, aged mice were subjected to local injection of the iron chelator DFO or the ferroptosis inhibitor ferrostatin-1 (Fer-1) into the tibialis anterior (TA) muscles. Following the treatment regimen, the mice were euthanized, and TA muscles were harvested. Notably, the administration of DFO or Ferr-1 significantly increased TA muscle mass in aged mice (Figure 6A) and improved muscle contractile properties, including 1/2 relaxation and specific twitch force (Figure 6B). Furthermore, grip strength (Figure 6C) and exercise performance, specifically running time and distance to exhaustion (Figure 6D), were markedly enhanced in aged mice following DFO or Ferr-1 treatment, suggesting that pharmacological inhibition of iron overload or ferroptosis effectively ameliorates muscle function in aged mice. Immunofluorescence staining was performed to identify muscle fiber types, including type I (blue), type IIa (green), and type IIb (red) fibers, to evaluate fiber-type switching following treatments. Compared with the untreated aged controls (Old), DFO and Ferr-1 treatments significantly reduced the proportions of type I and type IIa fibers while increasing the proportion of type IIb fibers in TA muscles of aged mice (Figure 6E). Additionally, the myofiber cross-sectional area (CSA) of the TA muscles was significantly enlarged in DFO- or Ferr-1-treated aged mice, indicating that sarcopenia-like muscle atrophy was effectively attenuated (Figure 6F,G). Moreover, the expression levels of muscle atrophy markers, namely, Fbxo32 and Trim63, were significantly downregulated in aged mice treated with DFO or Ferr-1. Corroborating these findings, genes associated with inflammation and fibrosis, such as interleukin (IL)-1β, tumor necrosis factor (TNF)-α, and collagen types I and III, which are known to be elevated in aged mice compared to young controls [15], exhibited a pronounced reduction in the TA muscles of treated aged mice (Figure 6H). Collectively, these results demonstrated that the DFO or Ferr-1 administration effectively alleviated skeletal muscle atrophy and improved muscle function in aged mice.

**Figure 7:**
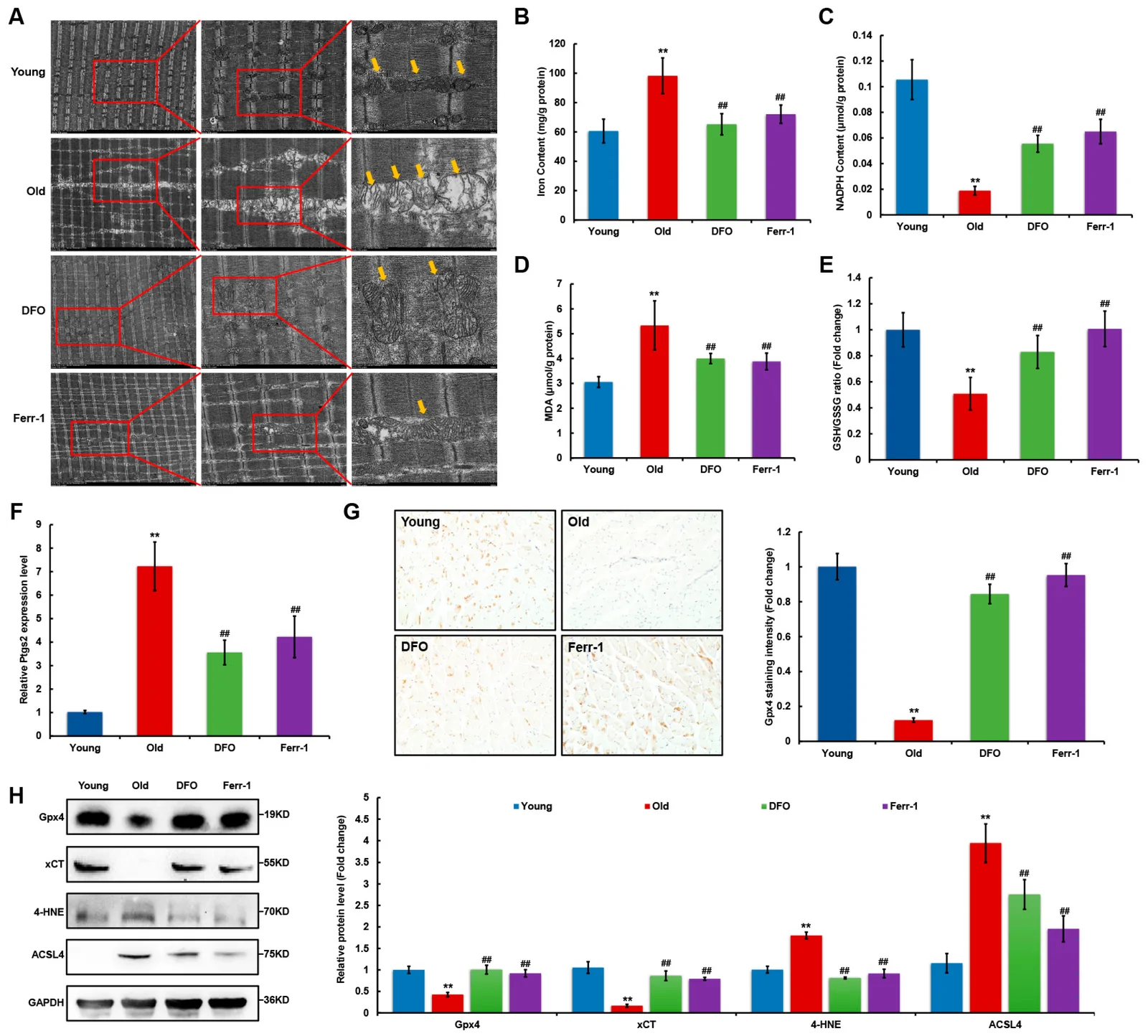
DFO and Ferr-1 alleviated ferroptosis in aged C57BL/6J mice. (**A**) Representative TEM images of TA muscles showing the ultrastructure of mitochondria in young and old C57BL/6J mice under different treatments (magnification: 3000×, 8000× and 20,000×; arrows indicate mitochondria). Bar graphs showing iron content (**B**), NADPH content (**C**), MDA (**D**), GSH (**E**), and Ptgs2 mRNA (**F**) levels in TA muscles of the young and old C57BL/6J mice under different treatments. ** *p* < 0.01 vs. the young group, ^##^ *p* < 0.01 vs. the old group. *n* = 6 per group. (**G**) Representative IHC images and quantitative analysis of Gpx4 staining in TA muscle sections of the young and old C57BL/6J mice under different treatments. ** *p* < 0.01 vs. the young group, ^##^ *p* < 0.01 vs. the old group. *n* = 7 per group. (**H**) Representative immunoblots and quantitative analysis showing the expression levels of Gpx4, xCT, 4-HNE and ACSL4 in TA muscles of the young and old C57BL/6J mice under different treatments. ** *p* < 0.01 vs. the young group, ^##^ *p* < 0.01 vs. the old group. *n* = 4 per group.

Morphological assessment of TA muscles by transmission electron microscopy (TEM) revealed that DFO or Ferr-1 treatment markedly ameliorated mitochondrial morphology. Consistent with previous studies demonstrating that ferroptosis is characterized by a transition from a large, compact mitochondrial architecture with intact cristae to a small, disorganized structure exhibiting reduced or absent cristae [32,33,34], the mitochondria in the TA muscles of aged mice treated with DFO or Fer-1 exhibited a restoration of their typical voluminous and compact morphology, coupled with intact cristae (Figure 7A), suggesting that both treatments effectively suppress ferroptosis in aged skeletal muscle. In addition, compared with the untreated aged control group, TA muscles from DFO- or Ferr-1-treated aged mice exhibited significant reductions in iron content, MDA concentration, and Ptgs2 mRNA expression, alongside a notable increase in NADPH and GSH levels (Figure 7B–F). Correspondingly, the protein levels of GPX4 and xCT were significantly upregulated, whereas ACSL4 and 4-HNE were markedly downregulated in the TA muscles of treated aged mice (Figure 7G,H), further corroborating the efficacy of DFO or Ferr-1 in mitigating age-related ferroptosis in skeletal muscle.

## 4. Discussion

Iron metabolism plays a pivotal role in the aging process. With age, systemic iron homeostasis becomes dysregulated, leading to iron accumulation in tissues such as the brain, liver, and other cellular niches [35]. Excess intracellular iron promotes the generation of reactive oxygen species (ROS) and lipid peroxidation, triggering ferroptosis—a form of iron-dependent cell death that has been linked to cellular senescence and age-related functional decline. Furthermore, senescent cells often exhibit persistently high levels of labile iron, which can both reinforce the senescence-associated secretory phenotype (SASP) and, paradoxically, render cells more susceptible to ferroptosis. Interventions that modulate iron levels—through chelation, dietary regulation, or targeting ferroptosis pathways—have shown promise in mitigating age-related tissue damage and extending health span in animal models [36,37].

Sarcopenia, characterized by age-related muscle atrophy, has also been reported to be closely associated with elevated iron content in skeletal muscles [38,39]. Aging muscles commonly exhibit molecular alterations, including decreased transferrin receptor 1 (TfR1) expression and increased accumulation of ferritin and non-heme iron, which collectively disrupt iron homeostasis and promote inflammation. Elevated serum ferritin or systemic iron levels have been shown to be negatively correlated with muscle mass and physical performance and positively associated with an increased risk of sarcopenia [12,40]. In our previous study, we demonstrated that iron overload induced ferroptosis in C2C12 cells and impaired their differentiation from myoblasts to myotubes. Furthermore, aged SAMP8 mice, a well-established model of sarcopenia, exhibited evidence of iron overload and ferroptosis in their skeletal muscles, supporting a pivotal role for iron metabolism in the pathogenesis of sarcopenia [15]. Nevertheless, the direct impact of iron dysregulation in the skeletal muscles of naturally aged wild-type mice remains largely unexplored.

Although oxidative stress and ferroptosis share common features, including ROS production and lipid peroxidation, they represent distinct biological processes. Oxidative stress is defined as an imbalance between oxidants and antioxidants; however, it does not necessarily cause cell death. In contrast, ferroptosis is a regulated form of cell death characterized by iron dependence and lipid-specific peroxidation. Key distinctions include the following: (1) ferroptosis strictly requires intracellular iron to drive the Fenton reaction and hydroxyl radical generation; (2) it specifically targets PUFA-containing phospholipids in cellular membranes; (3) GPX4 serves as the primary defense, and its loss is a ferroptosis hallmark; and (4) ferroptosis cells exhibit characteristic mitochondrial shrinkage with increased membrane density [11,37]. In our study, senescent myoblasts displayed TfR1 upregulation, decreased iron storage proteins (FTL, FTH), and increased Fe^2+^ accumulation. Mitochondrial morphology revealed characteristic ferroptosis-related alterations, GPX4 was downregulated, and both ferrostatin-1 and deferoxamine effectively rescued the aforementioned ferroptosis phenotypes. Collectively, these findings demonstrate that senescent myoblasts undergo ferroptosis rather than general oxidative stress-induced damage.

It is important to note that the high concentration of D-galactose (40 mg/mL) used in this study induces substantial cytotoxicity. While ferroptosis inhibitors (DFO and Ferr-1) significantly alleviated cell death, the recovery was partial. This suggests that under such severe stress conditions, other forms of cell death or nonspecific cellular stress pathways may also be activated alongside ferroptosis. Future studies using more specific inducers may help to further dissect the specific contribution of ferroptosis in senescent myoblasts.

As a pivotal link between iron homeostasis and ferroptosis, TfR1 serves as the rate-limiting step for iron uptake and critically regulates the labile iron pool. Its upregulation amplifies ROS and lipid peroxidation, thereby sensitizing cells to ferroptosis, whereas TfR1 knockdown or inhibition attenuates this process [41]. In the present study, we observed elevated TfR1 expression, alongside decreased expression of FPN1, FTL and FTH in D-gal-treated C2C1 cells, suggesting that senescence-induced iron overload in myoblasts is likely driven by increased iron influx coupled with decreased iron efflux and storage. A recent study indicated that TfR1 ablation in satellite cells impaired skeletal muscle regeneration by activating ferroptosis [42], a finding that aligns with the senescence-associated upregulation of TfR1 and increased ferroptosis observed in myoblasts in our current study. Therefore, we aimed to elucidate the factors and mechanisms underlying senescence-induced TfR1 upregulation. Furthermore, we discovered that 3-MA partially reversed TfR1 upregulation, suggesting that autophagy was involved in this process and that senescence-induced iron overload may be attributable to ferritin degradation resulting from excessive autophagy.

A specialized form of selective autophagy, ferritinophagy, specifically targets the iron-storage complex ferritin for lysosomal degradation [21]. The cargo receptor NCOA4 (nuclear receptor co-activator 4) binds the ferritin heavy chain (FTH1) and delivers the ferritin-NCOA4 complex to autophagosomes, where it is degraded, thereby releasing bio-available Fe^2+^ into the cytosol. Through this mechanism, NCOA4 serves as the pivotal molecular bridge that links the autophagic machinery to iron homeostasis [23,24]. In the present study, we demonstrated that NCOA4 was markedly upregulated, and its knockdown not only inhibited the reduction in FTH and decreased intracellular iron levels but also prevented ferroptosis in D-gal-induced senescent C2C12 cells. These findings indicate that NCOA4-regulated ferritinophagy contributes to senescence-induced iron overload and ferroptosis in myoblasts, representing one of the potential mechanisms underlying sarcopenia.

Mitochondria, serving as the central hub in ferroptosis, have been observed to exhibit characteristic morphological alterations, including organelle shrinkage, increased membrane density, loss or disappearance of cristae, and rupture of the outer membrane. These morphological changes are indicative of disrupted oxidative phosphorylation and elevated mitochondrial reactive oxygen species (mitoROS) production, which drive iron-dependent lipid peroxidation [33,43]. Two key mitochondrial iron-handling proteins regulate this process: mitoferrin-2 (Mfrn2) imports ferrous iron into the matrix, whereas mitochondrial ferritin (Ftmt) sequesters excess iron within the matrix [44]. Mfrn2-mediated iron import expands the labile redox-active iron pool, whereas Ftmt-mediated sequestration restricts iron-driven ROS generation [45]. In the present study, mitochondrial morphology underwent significant alterations in senescent myoblasts, and both MMP and ATP synthesis were reduced—phenotypic changes that were alleviated by DFO treatment. We observed elevated Mfrn2 and diminished Ftmt levels, indicating mitochondrial iron import. This imbalance likely contributes to mitochondrial dysfunction and commitment to ferroptosis.

As a small molecule ferroptosis inhibitor, ferrostatin-1 (Ferr-1) directly scavenges lipid radicals and blocks iron-catalyzed lipid peroxidation [46]. Deferoxamine (DFO), an iron chelator approved by the FDA, has been demonstrated to alleviate muscle wasting by restoring intracellular iron homeostasis [47,48]. In the present study, administration of DFO or ferrostatin-1 effectively suppressed iron accumulation and ferroptosis in senescent myoblasts while ameliorating muscle morphology and function in aged mice. These results suggest that targeting iron overload or ferroptosis represents a potential therapeutic strategy for sarcopenia, extending previous observations of the protective effects of DFO and ferrostatin-1 in dexamethasone-induced atrophy models to naturally aged skeletal muscle—a more clinically relevant setting.

## 5. Conclusions

**Figure 8:**
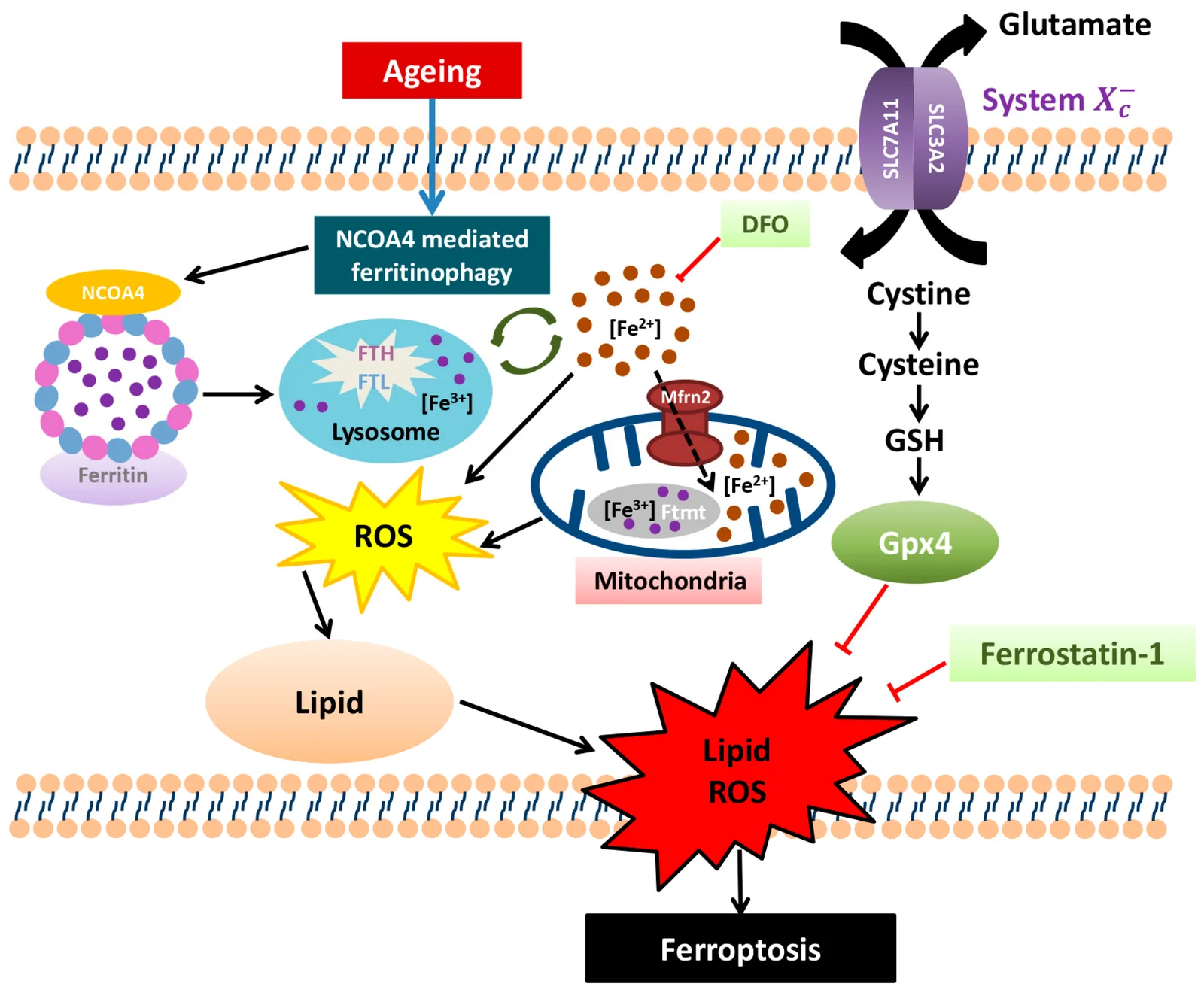
Schematic illustration of the mechanism of NCOA4-mediated ferritinophagy contributing to iron overload-driven ferroptosis in senescent myoblasts.

In summary, the present study suggests that iron overload and ferroptosis in senescent myoblasts may contribute to the pathogenesis of sarcopenia, and that treatment with the iron chelator DFO or the ferroptosis inhibitor Ferr-1 could attenuate these effects, potentially improving muscle quality and function in aged mice. Mechanistically, our findings indicate that aging may promote ferritinophagy through the upregulation of NCOA4, which could lead to the release of free iron and the subsequent activation of Mfrn2 on the mitochondrial membrane of myoblasts. This Mfrn2-mediated import of cytoplasmic free iron into mitochondria is likely to result in mitochondrial iron overload and excessive ROS production, eventually leading to lipid peroxidation and ferroptosis of myoblasts (Figure 8). While further studies are warranted to fully establish the causal relationships, this pathway likely represents one of the potential mechanisms underlying sarcopenia.

## Data Availability

All data have been included in the manuscript.

## Supplementary Materials

The following supporting information can be downloaded at https://www.mdpi.com/article/10.3390/cells15161434/s1, Figure S1: D-gal-induced C2C12 cell death at varying concentrations was partly reversed by DFO and Ferr-1; Figure S2: D-gal induced senescence in C2C12; Figure S3: NCOA4 mRNA and protein expression levels in D-gal-induced C2C12 cells after transfection with different NCOA4 siRNAs.
