# Supplementary material for: NCOA4-Mediated Ferritinophagy Contributes to Iron Overload-Driven Ferroptosis of Senescent Myoblasts in Mice
Source: Cells. 2026 Aug 10;15(16):1434. doi: 10.3390/cells15161434 (PMC13510329; doi:10.3390/cells15161434)
Supplement: Supplementary file 1 [file cells-15-01434-s001.zip › cells-4299453-supplementary.pdf]

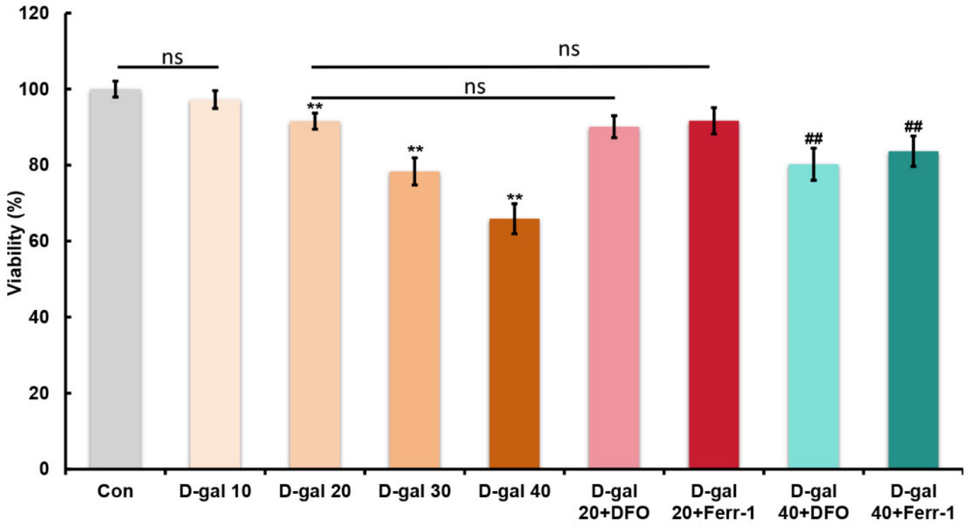

Supplementary Figure S1. D-gal-induced C2C12 cell death at varying concentrations was partly reversed by DFO and Ferr-1. Cell Viability of C2C12 was measured using the AlamarBlue assay after 48 hours of treatment with different concentration of D-gal in the absence or presence DFO or Ferr-1. Survival data were normalized to those of non-treated C2C12 cells. All data are presented as mean ± S.D. ns, $p \geq 0.05$; ** $p < 0.01$ *vs* control; $^{##}$ $p < 0.01$ *vs* D-gal (40mg/mL) treated group. n = 3 independent experiments.

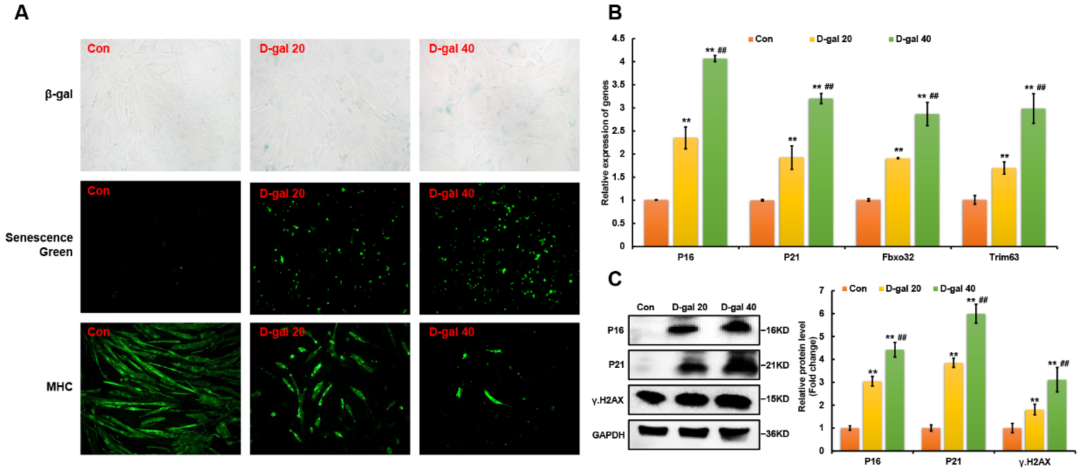

Supplementary Figure S2 D-gal induced senescence in C2C12. (A) Representative images of β-galactosidase (β-gal) and Senescence Green-stained myoblasts (magnification: 200x) and MHC stained myotubes (magnification: 100x) treated with varying concentrations of D-gal. (B) P16, P21, Fbxo32 and Trim63 mRNA levels in

myoblasts treated in the absence or presence of 20 mg/mL or 40 mg/mL D-gal (**p < 0.01 *vs* control group, ##p< 0.01 *vs* 20 mg/mL D-gal treated group). (C) Representative immunoblots and quantitative analysis showing the expression levels of P16, P21 and γ-H2AX in non-treated or 20 mg/mL and 40 mg/mL D-gal-treated myoblasts (**p < 0.01 *vs* control group, ##p< 0.01 *vs* 20 mg/mL D-gal-treated group). All data are presented as mean ± S.D. n = 3 independent experiments.

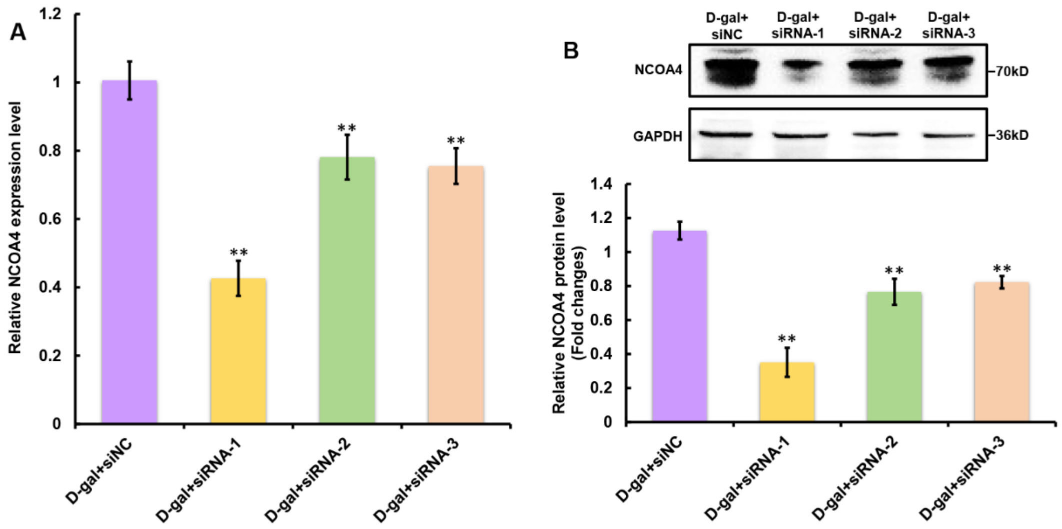

Supplementary Figure S3 NCOA4 mRNA and protein expression levels in D-gal-induced C2C12 cells after transfection with different NCOA4 siRNAs. D-gal treated C2C12 were transiently transfected with non-targeting control siRNAs (siNC) or three different NCOA4-targeting siRNAs (siRNA-1, -2, and -3) using Lipofectamine. Q-PCR(A) and western blotting (B) were used to assess the relative mRNA and protein expression levels of NCOA4, respectively. Due to the highest interference efficiency among the three NCOA4-targeting siRNAs, siRNA-1 was selected for the subsequent experiment. All data are presented as mean ± S.D. **p < 0.01 *vs* control. n = 3 independent experiments.
